# The Cytotoxic and Apoptotic Effects of *Scrophularia Atropatana* Extracts on Human Breast Cancer Cells

**DOI:** 10.15171/apb.2017.046

**Published:** 2017-09-25

**Authors:** Elham Safarzadeh, Abbas Delazar, Tohid Kazemi, Mona Orangi, Dariush Shanehbandi, Solmaz Esnaashari, Leila Mohammadnejad, Saeed Sadigh-Eteghad, Ali Mohammadi, Mehrdad Ghavifekr Fakhr, Behzad Baradaran

**Affiliations:** ^1^Immunology Research Center, Tabriz University of Medical Sciences, Tabriz, Iran.; ^2^Department of Pharmacognosy, Faculty of Pharmacy, Tabriz University of Medical Sciences, Tabriz, Iran.; ^3^Drug Applied Research Center, Tabriz University of Medical Sciences, Tabriz, Iran.; ^4^Neurosciences Research Center (NSRC), Tabriz University of Medical Sciences, Tabriz, Iran.

**Keywords:** Scrophularia atropatana, Breast cancer, Extract, Apoptosis, MCF-7, Cytotoxic

## Abstract

***Purpose:*** Breast cancer is the most frequent malignancy diagnosed in women both in developed and developing countries. Natural products especially those from herbal origin have high potential in producing drug components with a source of novel structures. The present study was designed to explore the cytotoxic effects and the cell death mechanism of Scrophularia atropatana extracts.

***Methods:*** MTT assay was employed to evaluate the cytotoxic activity of the extracts of S. atropatana on the MCF-7 as well as non-malignant cells. Furthermore, induction of apoptosis was evaluated by TUNEL assay, cell death detection ELISA, DNA fragmentation test, western blotting and Real Time PCR.

***Results:*** In vitro exposures of the MCF-7 cells with different concentration of S. atropatana extract significantly inhibited their growth and viability and induced apoptosis in the MCF-7 cells. Cleavage PARP protein, decrease in the mRNA expression levels of bcl-2 and increase expression of Caspase-3 and Caspase-9 mRNA, highlights that the induction of apoptosis was the main mechanism of cell death. Moreover the expression study of Caspase-9 mRNA showed that, the extracts have induced apoptosis via intrinsic mitochondrial pathway.

***Conclusion:*** Our results demonstrated that dichloromethane extract of Scrophularia atropatana has an apoptotic effects and it can be developed as anticancer agents.

## Introduction

Cancer is one of the most common causes of death worldwide. It is estimated that by 2030, there will be 21.4 million new cases of cancer and 13.2 million cancer deaths annually in the world.^1-3^ The most conventional strategies for cancer therapy involve surgery, radiotherapy and chemotherapy.^4,5^ Among these methods, chemotherapy is widely used for treatment of cancer. However, this therapy has serious side effects and complications.^2,3^ Hence, seeking for new therapeutic agents and effective therapies to prevent or control the complications and side effects of routine drugs is of a central importance.^1^ Natural compounds especially herbal medicines have a long history of use as medication for various diseases such as cancer.^3,6,7^ Many conventional drugs have been derived from herbal resources.^8,9^ According to the World Health Organization (WHO), herbal medicines account for approximately 80% of therapeutics in the developing countries.^10,11^ Herbal medicines are increasingly being investigated to overcome the side effects of conventional cancer treatments.^12,13^ Herbal therapies are more accepted among public and are believed to be safe and natural.^14,15^ Several studies suggest that, herbal medicines may halt the tumor promotion and progression and show an acceptable range of cytotoxic activities on cancerous cells without causing excessive damages to normal cells.^16,17^ Some of the compounds with natural origin such as taxol (from *Taxus brevifolia*), camptothecin (from *Camptotheca acuminate*), Decne, vinca alkaloids from Catharanthus roseus G. Don and podophyllotoxin from Podophyllum peltuturn L. are often used in oncology and act as antitumor agents.^3,18,19^ The most prominent marker of anticancer agents is induction of apoptosis.^20-22^ Hence, apoptosis is considered as an impotent key event in cancer chemotherapy.^18,23^ Therefore, the identification of medicinal plants which induce apoptosis is of central importance.^24-27^ *Scrophularia*, an important herbal medicine, is a genus of the family Scrophulariaceae which includes about 3000 species and 220 genera throughout the world.^28,29^ Since ancient times, some species of *Scrophularia* genus are being used for treatment of several ailments such as fever, erythema, eczema, skin inflammation, different types of dermatosis and also for cancer and wound healing.^30^ Herein, we report the cytotoxic and apoptotic effects of *Scrophularia atropatana* extracts on MCF-7 (breast carcinoma cell line) for the first time.

## Materials and Methods

### Preparation of extracts

*Scrophularia atropatana* was collected from Eastern Azerbaijan province, Iran. The herbarium voucher specimens (8962) were identified and deposited by the Herbarium of the Faculty of Pharmacy, Tabriz University of Medical Sciences. The aerial parts of the *S. atropatana* (leaves and stems) were washed thoroughly with distilled water and air-dried at room temperature for 2 weeks. Then, the samples were ground using a blender and stored in an airtight container. Extracts from the ground samples were obtained by Soxhlet apparatus using n-hexane, dichloromethane and methanol. *S. atropatana* extracts were concentrated by rotary evaporator (Heildolph, Germany) at 45 °C dried under reduced pressure. Then, 20 mg of each extract was separately re-suspended in 100 µL of Dimethyl Sulfoxide (DMSO) (Merck, Germany) and diluted with serum-free culture medium, RPMI-1640. Finally, the plant extracts were sterilized with 0.22 µm syringe filters (Nunc, Denmark) and stored at 4 °C as a stock solution for further biological assays.

### Cell Culture

The Human breast carcinoma cell line (MCF-7) and a normal control cell line (L929) were purchased from Pasteur Institute of Iran (National Cell Bank). The cells were grown in RPMI-1640 medium (Sigma, Germany) supplemented with 10% heat inactivated fetal calf serum (FCS) (Sigma, Germany), 100 Units/ml penicillin and 100 µg/ml streptomycin (Sigma, Germany) and maintained at 37 °C in a humidified atmosphere of 5% CO_2_. MCF-7 cells were maintained in their growing phase at 80% confluence with routine passage using 0.025% Trypsin-EDTA treatment. According to the aims of the study, the cells sub cultured into 75cm^2^ flasks, 6-well Plates or 96-well plates (Nunc, Denmark).

### Cell Cytotoxicity Assay

The effect of n-hexane, dichloromethane and methanol extracts of *S. atropatana* on cell growth was analyzed by the MTT method based on the ability of live cells to convert 3-(4,5-dimethylthiazolyl-2-yl)-2,5-diphenyltetrazolium Bromide (MTT) into Purple formazan by mitochondrial dehydrogenases. Briefly, cells in early log phase were trypsinized and cultured in 96-well plates with concentration of 10^4^ cells/well/200 µl and incubated overnight at 37°C and 5% CO2. Twenty-four hours later, the existing medium was replaced with fresh medium containing different concentrations of extracts (0, 100, 150, 200, 300, 400, 500 and 600 μg/mL). Furthermore, 0.2 % (v/v) DMSO (Merck, Germany) as a negative control and Taxol (Paclitaxel) as a positive control were considered for this assay. After 24, 36 and 48 h of treatments, 10 μl MTT (5 mg/mL) was added to each well and incubated for 4h in a humidified atmosphere at 37°C following the manufacturer’s instructions. These incubation times in the present study were calculated according to the previous study of Iloki Assanga et al and doubling time of the cell lines.^31-33^ The formazan crystals were solubilized with DMSO and 25𝜇L of Sorenson buffer. Then the absorbance at a wavelength of 570nm was measured using ELISA plate reader (Bio Teck, Germany). All experiments were performed in triplicates. The dose-response curve was plotted and IC50 value (the concentration that caused 50% of cell growth inhibition) was calculated. Data were normalized by setting the DMSO control to one.

### Trypan blue dye exclusion assay

10^4^ MCF-7 cells as well as L929 cell (as a normal control cell) were seeded in 96-plate and treated with 0, 100, 200 and 300 μg/mL concentration of dichloromethane and methanol extracts of *S. atropatana* in 0.2 % (v/v) DMSO for 24, 36, 48 and 72 hours. Subsequently, the cells were detached by adding 50 μl of 0.5 % trypsin/EDTA. Then 50 μl of the suspended cells was mixed with an equal volume of trypan blue and incubated for 3 min. Finally, 20 μl of this solution transferred to a hemocytometer and the numbers of viable and non-viable cells were counted under an inverted microscope. The viability was calculated as follows: viability (%) = (live cell count/total cell count) ×100

### Cell Death Detection

Cell death detection ELISA (Roche Applied Science, Germany), determinates the mono- and oligonucleosomes in the cytoplasmic fraction of cell lysates after induced cell death using mouse monoclonal antibodies against DNA and histones.^34^ Briefly, cells were incubated with the IC50 concentrations of methanolic and dichloromethane extract* S. atropatana*. DMSO and Taxol were used as negative and positive controls respectively. After 24 hours of incubation at 37°C, the culture supernatants were utilized for quantification of necrosis and cell lysates for apoptosis. The assay was performed according to the manufacturer’s instructions. The absorbance was measured using an ELISA plate reader at 405 nm and the percentage of apoptosis and necrosis obtained from the ratio of absorbance in the treated samples to that of the untreated controls.

### TUNEL assay

TUNEL assay (Terminal deoxynucleotidyl transferase dUTP nick end labeling) was carried out for detection of apoptosis in the treated cells. The *in situ* cell death detection kit (Roche Diagnostics GmbH, Germany) was employed for this purpose. This method is based on the presence of a multitude of DNA strand breakages during programmed cell death. Terminal deoxynucleotidyl transferase enzyme (TdT) adds secondarily conjugated dUTPs to the end nicks.^35,36^ Concisely, MCF-7 cells and L929 cell were seeded on chamber slides. After 24 h, the medium was replaced with fresh one. The cells were treated with IC50 concentration of *S. atropatana* dichloromethane extract. Following incubation, cells were washed with PBS and fixed in freshly prepared 4% (w/v) paraformaldehyde (pH 7.4) for 60 min at room temperature. Subsequently, cells were incubated with blocking solution (3% H_2_O_2_ in methanol) (Merck, Germany) at room temperature for 10 min to inactivate the endogenous peroxidases. The cells were washed once with PBS and permeabilized in 0.1% Triton X-100 in 0.1% sodium citrate for 2 min on ice. Then the fixed and permeabilized cells were incubated in TUNEL reaction mixture (containing TdT-enzyme and Biotinated-dUTP) for 60 min at 37°C in the dark. For negative control, label solution (without TDT) was used instead of reaction mixture. Cells incubated with DNase I (to induce DNA strand nicks) served as positive control. All cells were washed twice with PBS and incubated with streptavidin-HRP conjugate (50μL per specimen) for 30 min and then rewashed. The cells were incubated with diaminobenzidine solution (DAB) for 10 min in the dark place. Afterward, the stained cells (dark brown cells) were analyzed under inverted biological microscope.

### DNA Fragmentation

A key event during apoptosis is DNA laddering in which, DNA is degraded by caspase-activated DNase (CAD). Genomic DNA at inter-nucleosomal linkers is cleaved by CADs into nucleosomal units, which are multiples of about 180-bp fragments.^37,38^ To perform this experiment, 4 × 10^5^ cells/well were cultured in 6-well plates and treated with 300 and 600 μg/mL concentrations of dichloromethane extracts of *S. atropatana* for 24 hours. DMSO 0.2 % (v/v) was exploited as a negative control. After trypsinizing, the cells were harvested by centrifugation (1000 g, 5 min, 4°C) and washed with 1X PBS. Then the cells were lysed with 1 ml lysis buffer (5 mM Tris [pH 8.0] 20 mM EDTA, 0.5% Triton X-100) and incubated overnight at 56 °C (proteinase K, Thermo scientific). After 24 h, NaCl (5M) was added and the procedure was followed by phenol chloroform method. DNA was precipitated by ethanol (100%) and the pellet was air-dried at room temperature. Finally the DNA pellet was washed once with ethanol (70%) and dissolved in nuclease free distilled water. The extracted DNA was treated with l µl of RNase A (DNase free, Fermentas) and incubated for 30 min at 37 °C. Electrophoresis was carried out utilizing agarose gel (1.8%) and the results were subsequently visualized under UV light.

### Quantitative Real Time-PCR

Cells were seeded in 6-well plates and exposed to dichloromethane extract of *S. atropatana* in concentration of 300 and 600μg/mL for 12 h at 37°C. Following incubation cells were washed and were added RNX^TM^ –PLUS. Then the sample incubated with chloroform for 5 min on the ice and centrifuged. Transfer the aqueous phase to a fresh tube RNase free and were added isopropanol and incubate for 15 min at 4°C. Washed RNA pellet with 70% EtOH, dissolved the pellet in DEPC water and quantified extracted RNA by spectrometric assay. Then cDNA were synthesized using Revert Aid ^TM^ first strand synthesis kit (Fermentase, Canada). Synthesized cDNA was measured on a Corbett Rotor Gene 6000 real-time PCR detection system using a SYBR Green I PCR Master Mix (ABI, Foster City, USA). PCR cycling conditions were 95°C for 10 min as hold step, followed by 45 cycles of 95°C for 20 s, 60°C for 30s. β- actin was used as an internal reference. The sequence of primers were: β-actin: Forward: 5´TCCCTGGAGAAGAGCTACG 3´, Reverse: 5´ GTAGTTTCGTGGATGCCACA 3´, bcl-2: Forward: 5´ CCTGTGGATGACTGAGTACC 3´, Reverse: 5´ GAGACAGCCAGGAGAAATCA 3´, caspase 3: Forward: 5´ TGTCATCTCGCTCTGGTACG 3´Reverse:5´AAATGACCCCTTCATCACCA 3´. Caspase-9: forward: GCAGGCTCTGGATCTCGGC and reverse: GCTGCTTGCCTGTTAGTTCGC.

### Western blotting for assessment of Poly (ADP-ribose) polymerase (PARP) protein cleavage

MCF-7 cells were grown in 6-well plates and treated with dichloromethane extracts of *S. atropatana* and DMSO, as a control for 24h. After treatment, cells were lysed in RIPA Extraction Buffer (Thermo Scientific, Canada) and equal amounts of proteins (100 μg) from cell lysates were subjected to sodium dodecyl sulfate– polyacrylamide gel electrophoresis (SDS- PAGE). Subsequently, blotting onto polyvinyl-difluoride (PVDF) membrane (Roche Diagnostics GmbH, Germany) was performed in 150 mA for one hour. The membrane was subsequently blocked with 4% skim milk, washed and incubated with specific primary antibodies of a mouse anti-PARP monoclonal antibody and anti-β-actin as a normalizing control (Roche Diagnostics GmbH, Germany) for overnight. Then the membrane was probed with horseradish peroxidase-conjugated secondary antibody. Protein detection was carried out by exposing the membrane to ECL western blotting detection system (Amersham Phamacia Biotech Inc, USA).

#### Statistical analysis

All statistical analyses were carried out using Graph Pad Prism 6.01 software (Graph Pad Software Inc., San Diego, CA, USA) and statistical signiﬁcance of diﬀerences were analyzed using two-way ANOVA test. Each experiment was performed in triplicates (n = 3) and Data are presented as the means ± S.D. The criterion for statistical significance between groups were considered as P<0.05.

## Results

### S.atropatana treatment inhibited MCF-7 cells viability and proliferation

**Figure 1:**
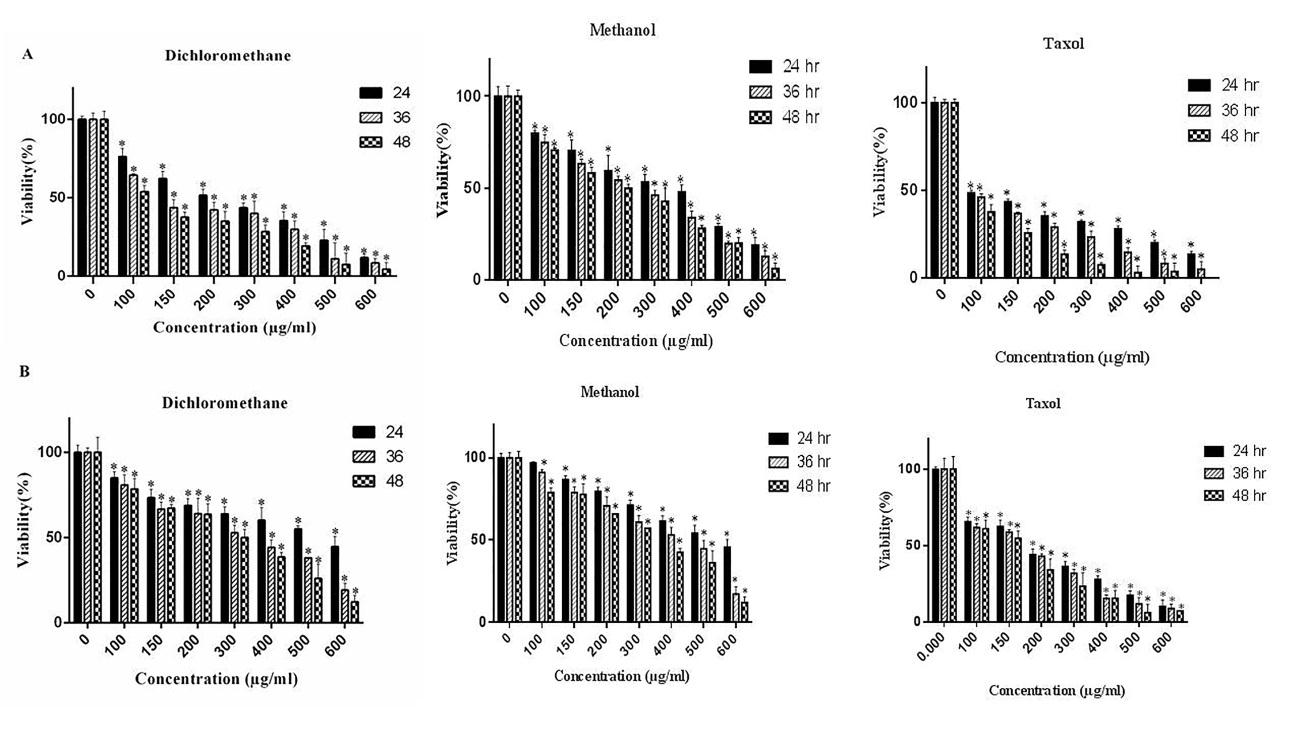
Cytotoxic effect of *S. atropatana* extract in A.MCF-7 cells, B.L929 cells Experiment was carried out in triplicates (n=3) and values are presented as the mean ± SEM.

**Figure 2:**
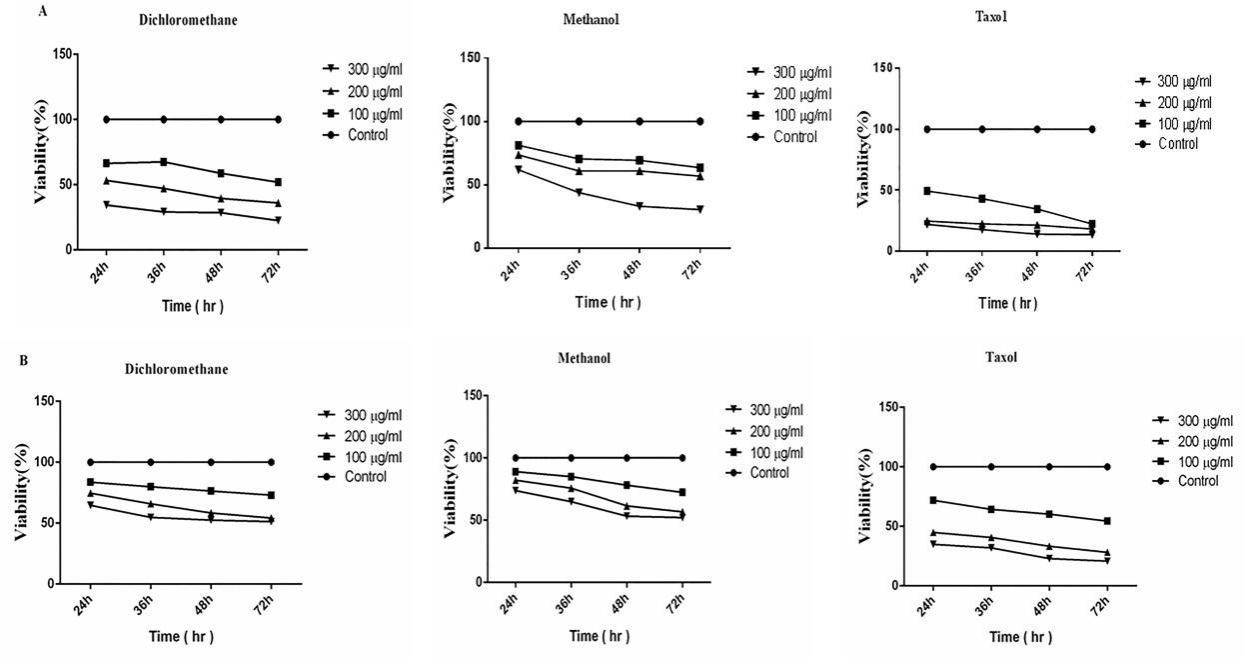
Viability of A.MCF-7 cells, B.L929 cells treated with various concentrations of *S. atropatana* (100, 200 and 300 μg/ml) for 24, 36, 48, and 72 hours.

To evaluate the cytotoxic effects of *S. atropatana* n-hexane, dichloromethane and methanol extracts on the growth of MCF-7 breast cancer cell line and L929, the cells were treated with different concentration of the extracts and then analyzed by MTT and trypan blue assay. As shown in Figure1, compared with untreated cells, dichloromethane and methanol extracts of *S. atropatana* significantly suppressed MCF-7 cell growth in a time and dose dependent manner, whereas the cytotoxic effects was not observed in the cells treated with n-hexane extract. Based on MTT results, the IC50 (50% growth inhibition) values of dichloromethane and methanolic extracts are shown in Table 1. According to the outcomes, dichloromethane extract exhibited a higher cytotoxic activity on MCF-7 cells than methanolic extract. Moreover, interestingly the cytotoxic effects of dichloromethane and methanol extracts of *S. atropatana* on the MCF-7 cells were significantly higher than on the L929 cells (p<0.05). In addition, dye exclusion assay was utilized for assessment of viability in MCF-7 and L929 cells treated with dichloromethane and methanol extract of *S.atropatana*. Taxol (paclitaxel) was employed as a positive control at the same concentration. Microscopic cell count using a hemacytometer indicated a significant decrease in the number of viable MCF-7 cells in the treated cells compared to untreated ones. As a result, the dichloromethane extract was more cytotoxic compared with methanolic extract (p<0.05) Figure 2.

**Table 1:** The IC50 values of dichloromethane and methanolic extracts in MCF-7 and L929cells.

**IC50 µg/ml**						
	**24 h**		**36 h**		**48 h**	
Cell line	MCF-7	L929	MCF-7	L929	MCF-7	L929
Dichloromethane	223.0	557.0	153.9	303.8	114.7	264.3
Methanol	289.9	543.3	226.6	370.2	197.9	321.0

### S. atropatana treatment induced apoptosis in MCF-7 cells

**Figure 3:**
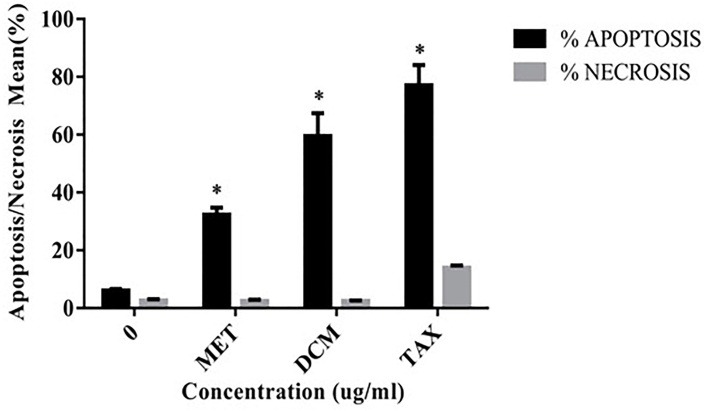
The percentages of apoptosis and necrosis by Cell Death assay in MCF-7 cells. Data are presented as means ± S.E. *: p < 0.05 was considered as significant statistical difference.

**Figure 4:**
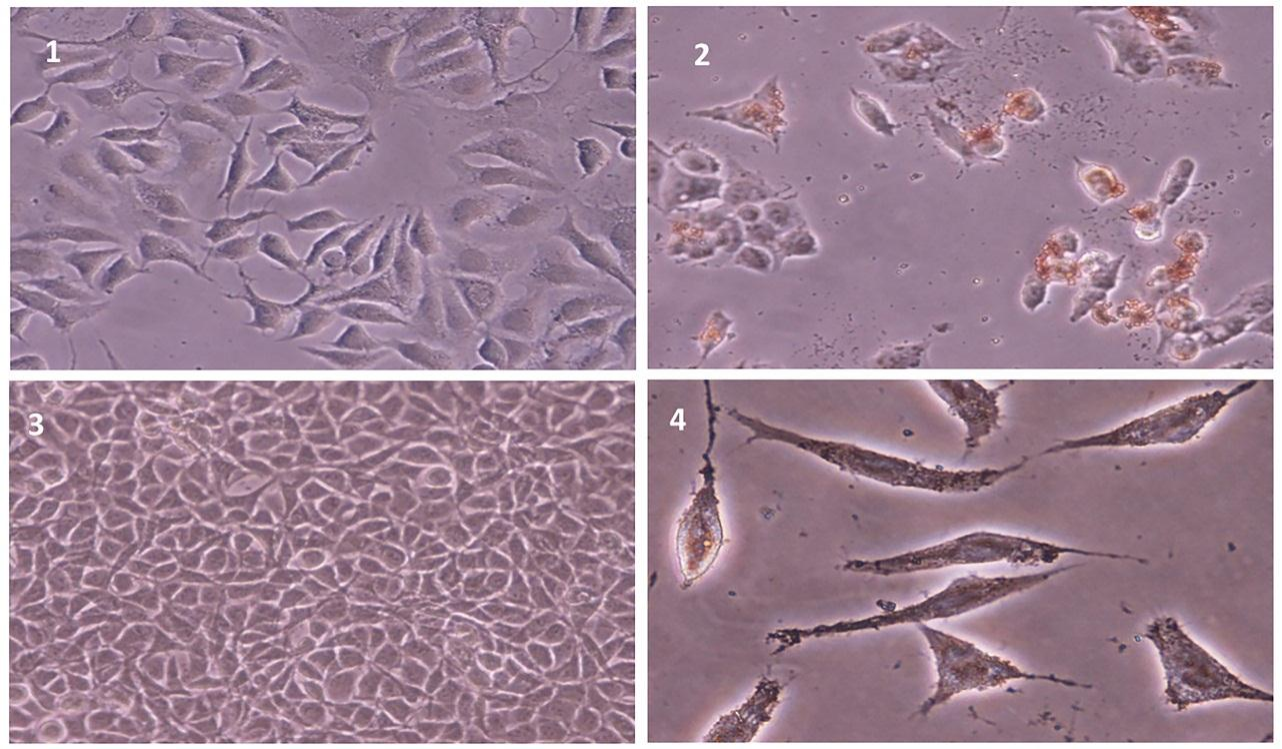
Apoptotic effects of *S. atropatana* on MCF-7 cells observed by TUNEL assay. (1) MCF-7 control, (2) treated MCF-7 (3) L929 control and (3) treated L929. Arrows indicate apoptotic cells.

Cell death ELISA kit, was used to assess apoptosis or necrosis occurrence in cells treated with dichloromethane and methanol extracts. Taxol was used as positive control for induction of apoptosis. After 24 h of treatment with IC50 concentrations of *S. atropatana* extracts, apoptosis values for dichloromethane extract and methanol extract in MCF-7 cells were 51.21% and 30.72% respectively (Figure 3). The acquired data indicated that, dichloromethane extract exhibits more apoptotic activity than methanol extract on the studied cells. Therefore, the next experiments were performed relying on dichloromethane extract. In addition, TUNEL test was performed to identify presence of DNA strand breaks and confirm the induction of apoptosis in MCF-7 cells exposed to dichloromethane extract *S. atropatana*. As shown in Figure 4, following 24 h of exposure to IC50 concentrations of the extracts despite the normal cells, apoptotic cells developed brown precipitates.

### S. atropatana treatment of MCF-7 cells induced DNA fragmentation

**Figure 5:**
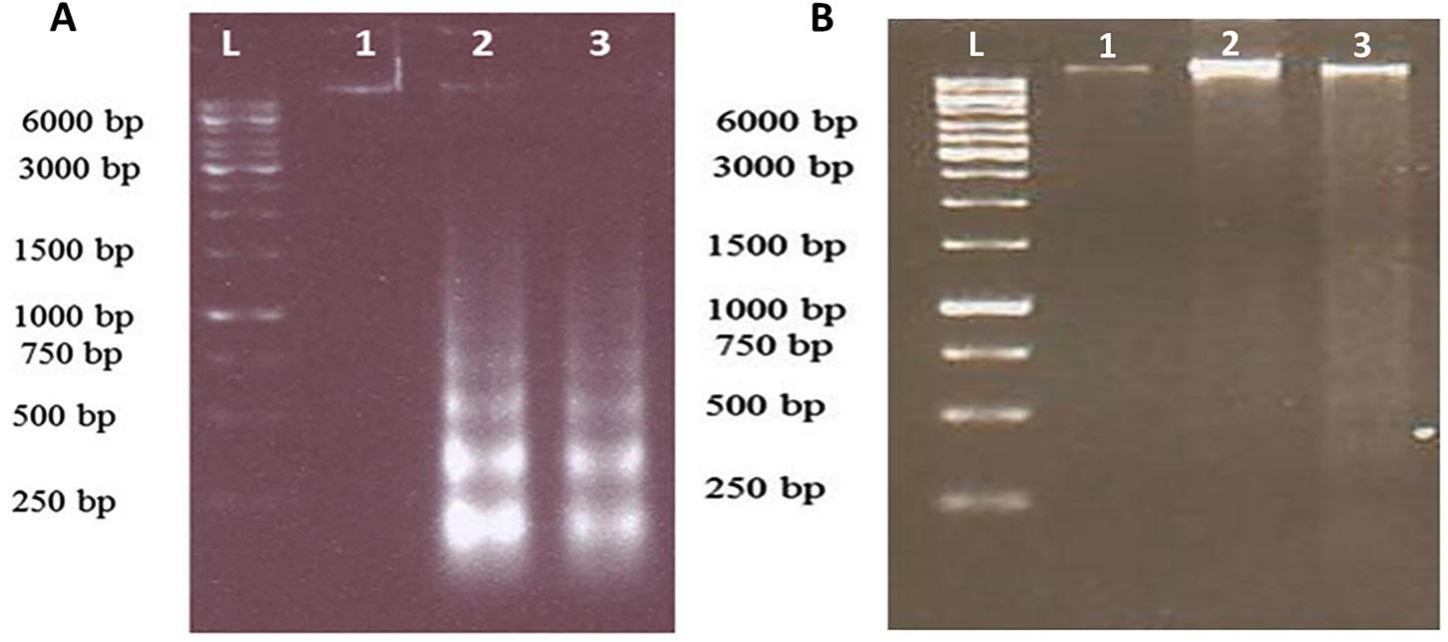
DNA fragmentation assay in MCF-7 cells(A) and L929 cells (B) treated with S. atropatana for 24 hr. (1) control,( 2) 300 µg/mL, (3) 600 µg/mL and L= size Ladder. DNA fragments were separated on 1.8% agarose gel electrophoresis.

A key feature of apoptosis is DNA fragmentation via nuclease activity of caspase-3 protein. Dichloromethane extract of *S. atropatana* was able to form apoptotic DNA ladders in MCF-7 cells after 24 h of treatment with the concentration of 300 and 600𝜇g/mL (Figure 5). Moreover, DNA fragmentation was not observed in L929 cells. The results further confirmed that, *S. atropatana* extract could induce apoptosis on tumor cells.

### S. atropatana treatment of MCF-7 cells changed the expression of apoptotic genes

**Figure 6:**
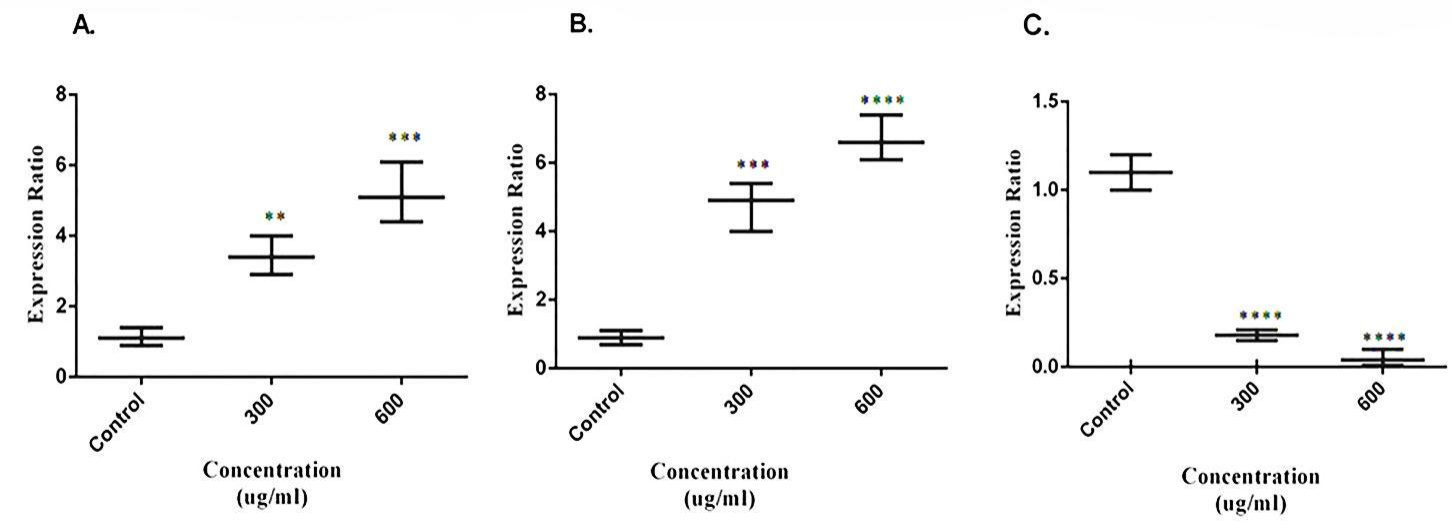
Effects of dichloromethane extract of S. atropatana on A. Caspase-3, B. Caspase-9 and **C.** Bcl-2 mRNA expression in MCF-7 cells at 24 h. Relative expression was acquired by qRT-PCR using 2(-ΔΔCt) method. The results are presented as mean ± SD (n = 3); ****p<0.00001 versus control ***p<0.0001 versus control.

Changes in the expression level of apoptosis-related genes, bcl-2 (anti apoptotic gene), caspase 3 (pro-apoptotic gene) and caspase 9 following the treatment with dichloromethane extract of *S. atropatana* was investigated using real time PCR. According to the data, bcl-2 expression was decreased by 6.25 and 22.22 fold in 300 and 600 µg/ml concentrations respectively compared to the untreated control cells, whereas a noticeable increase was detected in caspase 3 expression. The rate of this increase was 3.02 and 4.58 fold in 300 and 600 µg/ml groups respectively. Caspase 9 also indicated a 5.3 and 7.3 fold increases in 300 and 600 µg/ml groups respectively (Figure 6).

### S.atropatana treatment of MCF-7 cells induced PARP cleavage

**Figure 7:**
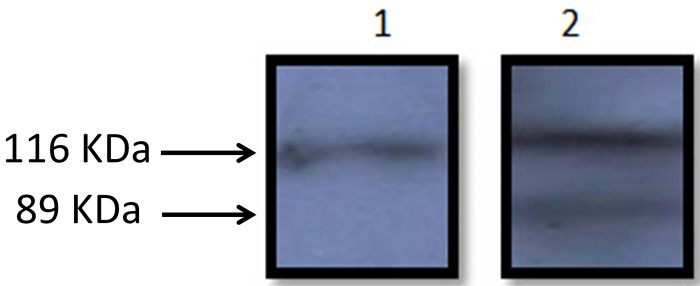
PARP molecule (116 kDa) cleavage to 89 kDa and 24 kDa fragments by activated caspase-3. (1) Control, (2) treated

During programmed cell death, the intact PARP molecule (116 kDa) is cleaved into 89 kDa and 24 kDa fragments by activated caspase-3.^39^ After 24 h of treatment with *S. atropatana* dichloromethane extract, the PARP molecule cleavage was detected in MCF-7 cells by western blot analysis. However, no cleavage of PARP molecule was observed in untreated control cells (Figure 7).

## Discussion

High prevalence of cancer makes it the second mortality cause following cardiovascular diseases. Nowadays, because of the priority and importance of neoplasia, numerous studies have focused on the introduction of safe and effective compounds for the treatment of various types of cancer. In this regard, the mechanisms involved in carcinogenesis has a critical role in developing the methods for neoplasm treatment.^3,40^ Among these compounds, drugs with herbal origin are the most popular. Because, these products are more cost effective, available and exert fewer side effects.^41^ Consequently, targeting the mechanisms which cause tolerance to death stimuli and apoptosis could be a suitable strategy in cancer treatment.^20^ Different studies have shown that, herbal medicines have different functional mechanisms, but inducing apoptosis is a common feature among them.^3^ Most synthesized anti carcinogenic drugs such as; taxol which used in treatment of breast and ovarian cancers have herbal origin.^3^

Numerous studies have shown the cytotoxic and anti-tumor effects of different species of Scrophularia genus. A study has shown that *Scrophularia lucida* has remarkable cytotoxic and apoptotic effects on HL-60 leukemic cells. It has been reported that methanolic extract of *S. lucida* leads to cell cycle blockage in G2-M phase, inhibiting activation of caspase-3 and breaking down PARP protein into 89 kDa compartments.^42^ In another study, the cytotoxic effects of *Scrophularia striata* on astrocytes (1321) has been assessed and shown that it had significant suppressing effects on the growth and replication of these cell types.^28^ Another study showed that methanolic and dichloromethanolic extracts of *Scrophularia oxysepala* suppresses the growth of MCF-7 cells by apoptosis induction.^43^ In the present study, the cytotoxic effect of n-hexane, dichloromethanolic and methanolic extracts of *S. atropatana* on MCF-7 and L929 cells has been assessed. The ability of elimination of tumor cells is one of the chemotherapeutic drug characteristics. The results of this study showed that dichloromethanolic and methanolic extracts of *S. atropatana* had significant cytotoxic effect on tumor cells but there was no significant activity in L929 normal cells. Nontoxic properties of these extracts on normal cells, makes them as suitable candidates for future in vivo studies. Moreover, N hexane extract did not indicate noticeable impacts on cancerous and normal cells. As shown in Figure1, the toxicity of the extracts is comparable with that of Taxol chemotherapeutic drug. Furthermore, the cytotoxic effect of dichloromethanolic and methanolic extracts was time and concentration dependent. It means that, by enhancing the concentration of extract and time of exposure, the cytotoxic effect on MCF-7 cells was increased. Also the comparison of IC50 showed that dichloromethanolic extract had greater effect on MCF-7 tumor cells than methanolic extract, suggesting that dichloromethanolic extract contain high level of active cytotoxic components. Cytotoxic effects are not solely enough for herbal extracts to be considered as antitumor agent. Beside this feature, chemotherapeutic extracts should also induce apoptosis. Microscopic observations showed that, the exposure of MCF-7 cells to dichloromethanolic and methanolic extracts led to morphological alteration and apoptotic bodies' formation. For evaluating the apoptosis induction, ELISA cell death assay was utilized. According to the results, dichloromethanolic and methanolic extracts exert apoptotic characteristics. Data analysis suggested that, these extracts had substantial apoptotic effects on MCF-7 cells compared to taxol. DNA fragmentation as one of the hallmarks of apoptosis could be demonstrated by TUNEL and DNA fragmentation test. The results of these tests were in conformity with earlier tests. In order to detect antitumor mechanism(s) of *S. atropatana*, the expression rate of genes involved in apoptosis such as caspase 3, caspase 9 and BCL-2 were assessed by real time PCR. CASP 3 and CASP 9 genes belong to cysteine- aspartic acid protease family play a central role in the induction of apoptosis. BCL-2 is a regulatory protein that as an oncogene has anti apoptotic effects. The increased expression of CASP 9 gene in the treated cells could be regarded as an indicator of intrinsic (mitochondrial) apoptosis. Data analysis showed that following the treatment of MCF-7 cells, the expression of caspase 3 and caspase 9 mRNAs were increased significantly and expression of bcl-2 gene decreased in contrast to the untreated controls. On the other hand Caspase-3 activity could result in the breakdown of different proteins including PARP. According to western blot results, PARP protein was fragmented to 89 kDa and 24kDaproteins in size demonstrating the occurrence of apoptosis in treated cells. Consequently, the aforementioned features prove the occurrence of apoptosis in the cells exposed to *S. atropatana* extracts.

## Conclusion

The treatment of MCF-7 cells with dichloromethanolic and methanolic extracts of *S. atropatana* resulted in morphological changes, cytotoxic and apoptotic effect on this tumor cells while there was no significant inhibitory effect on normal cells. Induced cytotoxic effects were related to exposure time and concentration of the extract, so that by enhancing the time and concentration the viability of cells declined. These findings suggested that dichloromethanolic extracts of *S. atropatana* may contained more bioactive components compare to methanolic and n hexane extracts. According to these results, dichloromethanolic extract of *S. atropatana* can be a proper candidate for extraction of antitumor component.

## Acknowledgments

The authors wish to thank the Immunology Research Center of Tabriz University of Medical Sciences, for supporting the research (Grant#91/61).

## Ethical Issues

Not applicable.

## Conflict of Interest

The authors declare no conflict of interests.
